# Hierarchical Structure of Gold and Carbon Electrode for Bilirubin Oxidase-Biocathode

**DOI:** 10.3390/bios13040482

**Published:** 2023-04-17

**Authors:** Yuto Nakagawa, Seiya Tsujimura, Marc Zelsmann, Abdelkader Zebda

**Affiliations:** 1 Faculty of Pure and Applied Sciences, University of Tsukuba, 1-1-1 Tennodai, Tsukuba 305-8573, Ibaraki, Japan; 2 Japanese-French Laboratory for Semiconductor Physics and Technology (J-F AST)–CNRS–Université Grenoble Alpes–Grenoble, INP–University of Tsukuba, 1-1-1 Tennodai, Tsukuba 305-8573, Ibaraki, Japan; 3 Laboratoire des Technologies de la Microélectronique, LTM-CNRS-UJF, CEA-LETI, 17 av. des Martyrs, 38054 Grenoble, France; 4 UGA-Grenoble 1/CNRS/INSERM/TIMC-IMAG UMR 5525, 38000 Grenoble, France

**Keywords:** dynamic hydrogen bubble templating, oxygen reduction, air biocathode, bilirubin oxidase, hierarchical porous gold structure

## Abstract

Biofuel cells (BFCs) with enzymatic electrocatalysts have attracted significant attention, especially as power sources for wearable and implantable devices; however, the applications of BFCs are limited owing to the limited O_2_ supply. This can be addressed by using air-diffusion-type bilirubin oxidase (BOD) cathodes, and thus the further development of the hierarchical structure of porous electrodes with highly effective specific surface areas is critical. In this study, a porous layer of gold is deposited over magnesium-oxide-templated carbon (MgOC) to form BOD-based biocathodes for the oxygen reduction reaction (ORR). Porous gold structures are constructed via electrochemical deposition of gold via dynamic hydrogen bubble templating (DHBT). Hydrogen bubbles used as a template and controlled by the Coulomb number yield a porous gold structure during the electrochemical deposition process. The current density of the ORR catalyzed by BOD without a redox mediator on the gold-modified MgOC electrode was 1.3 times higher than that of the ORR on the MgOC electrode. Furthermore, the gold-deposited electrodes were modified with aromatic thiols containing negatively charged functional groups to improve the orientation of BOD on the electrode surface to facilitate efficient electron transfer at the heterogeneous surface, thereby achieving an ORR current of 12 mA cm^−2^ at pH 5 and 25 °C. These results suggest that DHBT is an efficient method for the fabrication of nanostructured electrodes that promote direct electron transfer with oxidoreductase enzymes.

## 1. Introduction

Biofuel cells (BFCs) with enzymatic electrocatalysts have attracted considerable attention, especially as power sources for wearable devices, owing to their ability to generate electricity from sugars and organic acids under very mild conditions [1]. BFCs typically use oxygen as an oxidant fuel at the cathode owing to its ready availability and high potential [2] Mano and de Poulpiquet, 2018). Bilirubin oxidase (BOD) from *Myrothesium verrucaria* is currently the most widely used biocatalyst for the oxygen reduction reaction (ORR) because it shows high activity at a neutral to slightly acidic pH and allows a low overpotential for the electrochemical ORR [3,4] Tsujimura et al., 2001; Mano and Edembe, 2013). BOD is a multi-copper oxidase containing four copper atoms, which can be categorized as one of three types (T1, T2, and T3) according to their magnetic and spectroscopic properties. The electrons are received from the electrode at the T1 copper site and passed to the T2–T3 copper cluster, where oxygen is reduced to water [5]. The T1 copper site on the surface of the BOD enzyme facing the electrode surface is located near to the electrode surface, and thus can receive electrons directly from the electrode. We used MgO template carbon (MgOC) as a scaffold for BOD to increase the concentration of BOD at the electrode surface [6,7]. The MgOC pore size can be easily controlled by adjusting the size of the MgO template [4]. Electrodes with macropores (between 150 and 200 nm in diameter) are more desirable than those with mesopores (between 10 and 40 nm in diameter) because the larger pore size allows the enzyme to more easily diffuse throughout the porous carbon layer, thereby improving its distribution and forming an adsorbed monolayer on the electrode [8]. Moreover, the larger pores allow O_2_ and electrolyte ions to diffuse more easily through the interconnected pores of the porous carbon layer; however, the efficiency of the current generation (current/enzyme loading) decreases with increasing pore size owing to a reduction in both the effective surface area and the number of T1 copper sites facing the electrode surface. The ratio of the specific and geometric surface areas can be increased by increasing the thickness of the carbon layer; however, a thick carbon layer impedes the mass transfer of O_2_ and ions to the surface enzyme, thereby reducing the current production efficiency. The operation of a BFC is typically limited by the ORR at the cathode owing to the limited O_2_ supply, which, in turn, is caused by its low solubility in water and slow diffusion in the solution [9].

Air-diffusion-type BOD cathodes have recently attracted significant attention as a means for overcoming the drawbacks of a limited O_2_ supply using the air O_2_ transport process, which occurs in a two-step process as follows: mass transfer of O_2_ from the air into the gas diffusion layer (GDL) and diffusion through the GDL, followed by dissolution into the solvent around the BOD, and subsequent diffusion through the solvent [4,7,9,10]. To overcome the low solubility of O_2_ in aqueous solutions via modification of the electrode with BOD, the specific surface area of the porous electrode should be as large as possible, while the solvent layer around the enzyme should be as thin as possible to increase the electrochemically active enzyme on the carbon surface without affecting mass transfer. Increasing the thickness of the carbon layer restricts the diffusion of ions and oxygen, while a non-uniform distribution of the enzyme reduces catalytic performance toward the ORR. Therefore, it is desirable to further develop the hierarchical structure of porous electrodes with highly effective specific surface areas [8,11].

**Figure 1:**
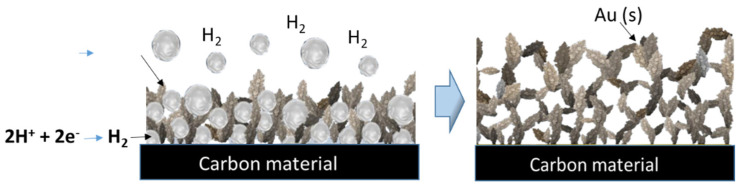
Schematic of the synthesis of a gold nanostructure on carbon using hydrogen bubbles. On the left: gold electrodeposition and protons’ reduction to hydrogen gas; on the right: porous gold nanostructures obtained via electrodeposition.

In this study, we placed a porous gold structure over the MgOC layer to increase the effective specific surface area without inhibiting the mass transfer. Gold is typically deposited onto surfaces via the reductive deposition of gold ions using a reducing agent [6,12] or electrochemical techniques [13]; however, controlling the pore size and structure of the deposited gold using these techniques remains a significant challenge. To achieve better control over the gold deposition, we fabricated a porous gold structure on top of a carbon layer using dynamic hydrogen bubble templating (DHBT), wherein the co-reduction of gold ions to gold metal and protons to H_2_ gas occurred simultaneously (Figure 1). The H_2_ gas acted as a template; thus, gold was deposited on the surface where H_2_ gas was not present. The porous structure was controlled by varying the Coulomb number and current density (i.e., the generation rate of H_2_ gas bubbles). The gold surface was further modified with a negatively charged aromatic thiol, which had a strong and specific interaction with the active site of BOD, to increase the number of T1 sites in BOD facing the electrode surface.

## 2. Materials and Methods

### 2.1. Materials

BOD (*Myrothecium verrucaria*, 2.0 U mg^−1^, Amano Enzyme Co., Ltd., Nagoya, Japan) was used without further purification. MgOC, with an average pore size of 200 nm, was purchased from Toyo Carbon Co., Ltd., Tokyo, Japan. The GDL was donated by the Freudenberg group (H23C6, Germany). Tripotassium citrate monohydrate, sulfonic acid, hydrochloric acid, 1-methyl-2-pyrrolidone (NMP), tetrabutylammonium bromide (TBAB), and ethanol were purchased from Fujifilm Wako Pure Chemical Industries Ltd., Tsukuba, Japan. 2-Mercaptobenzoic acid (2MBA) was procured from Tokyo Chemical Industry Co., Ltd., Tokyo, Japan. Tetrachloride gold oxide (III) hydrate, Nafion^®^ perfluorinated resin solution (5%), and 4-mercaptobenzoic acid (4MBA) were purchased from Sigma-Aldrich, Missouri, USA. Poly(vinylidene fluoride) (PVDF, 9305, 5 wt% in NMP) was donated by Kureha Co., Ltd., Tokyo, Japan.

### 2.2. Fabrication of the Porous Electrode

A mixture of MgOC (40 mg) and PVDF binder (0.2 mL, 5 wt% in NMP) was ultrasonicated for 1 min (UH-50, SMT Co., Ltd., Tokyo, Japan), and NMP (0.4 mL) was added to the obtained homogeneous dispersion. The MgOC ink was dropped on the GDL (0.25 µL cm^−2^) and dried at 60 °C for 12 h to form the MgOC electrode. Potentials of 2.0 V and −3.0 V (vs. saturated calomel electrode (SCE)) were applied for 120 s each in 100 mM citrate buffer solution (100 mM) to hydrophilize and clean the prepared MgOC electrode. Subsequently, a potential of −3.0 V was applied via chronoamperometry (CA) in a HAuCl_4_ solution (1 mM) up to the required electric charge density of *Q* C cm^−2^. The reduction of gold ions and the electrolysis of water occurred simultaneously, and hydrogen bubbles were generated on the working electrode. The prepared electrodes were described as MgOC-Au_X_ electrodes, based on the electric charge density, *Q* (*Q* = X C cm^−2^, where X = 1, 10, 15, and 20), whereby the gold was deposited. Finally, the carbon structures were examined using field-emission scanning electron microscopy (FE-SEM; SU-8020, Hitachi, Japan).

### 2.3. Thiol Modification

2MBA and 4MBA were dissolved in HCl solution (pH 2.0) at 5 mM (note that they were not completely dissolved). The electrodes with a deposited gold nanostructure were immersed in the thiol-containing HCl solutions for 12 h. Thereafter, the electrodes were carefully washed with distilled water and ethanol.

### 2.4. BOD Modification

A BOD solution (15 mg mL^−1^) was prepared in citrate buffer (100 mM, pH 5.0), to which 25 µL mL^−1^ of Nafion TBAB (100 mg mL^−1^) was added with stirring. Nafion TBAB was prepared according to an established procedure [1]. A drop of this Nafion TBAB solution (200 µL) was applied to the MgOC-Au electrode and dried under reduced pressure at 25 °C.

### 2.5. Electrochemical Measurement

Electrochemical measurements were performed using MgOC as a working electrode in a three-electrode system. SCE and platinum wire were used as the reference and counter electrodes, respectively. The electrode was evaluated using citrate buffer (100 mM, pH 5.0) as the electrolyte. The O_2_ gas was bubbled through the electrolyte to ensure the O_2_ saturation. Cyclic voltammetry was performed between 0 and 0.6 V (vs. SCE) at a scan rate of 2 mV s^−1^ and CA was conducted at a potential of 0.2 V.

## 3. Results and Discussion

### 3.1. Gold Modification on MgOC Electrode

**Figure 2:**
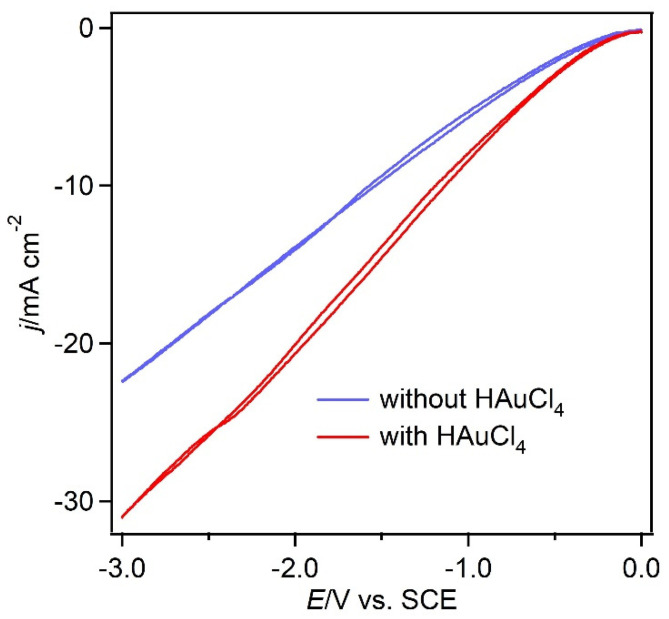
CVs recorded using MgOC in HCl solution (1 M) in the absence (blue) and presence (red) of HAuCl_4_ (1.5 mM) at a scan rate of 10 mV s^−1^ at 25 °C.

Cyclic voltammograms (CVs) of MgOC were recorded at 10 mV s^−1^ in HCl solutions (1 M) with and without Au ions (Figure 2). Reduction currents were observed at −300 mV in both cases and increased considerably from −650 mV. Thus, the electrochemical reduction of Au ions began at approximately −650 mV; however, the generation of hydrogen bubbles became favorable below −300 mV, which was confirmed visually. The electrodeposition of gold on the MgOC surface in the presence of hydrogen bubbles can therefore be achieved at potentials below −650 mV vs. SCE.

**Figure 3:**
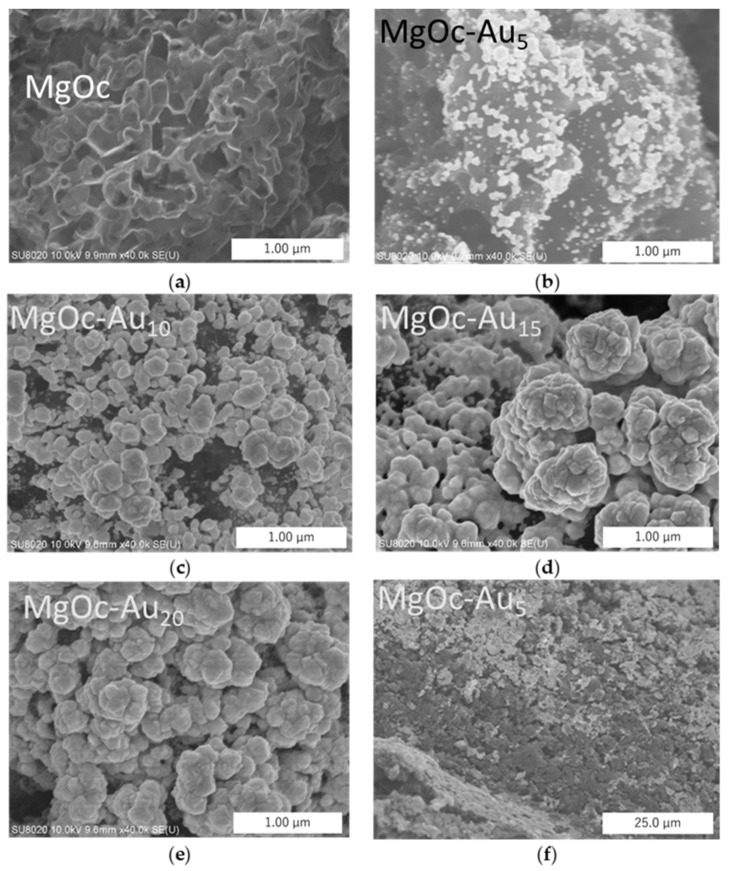
FE-SEM images of MgOC: (**a**) MgOC modified with gold nanostructures; (**b**) MgOC-Au_5_; (**c**) MgOC-Au_10_; (**d**) MgOC-Au_15_; (**e**) MgOC-Au_20_; and (**f**) cross-section of MgOC-Au_5_.

The reduction of Au^3+^ and the generation of hydrogen via proton reduction occurred simultaneously in HAuCl_4_ solution (1.5 mM) at an applied potential of −3.0 V. Both of these processes contributed to the overall current density. The amount of gold deposited on the carbon surface was modulated by adjusting the electric charge density *Q* (C cm^−2^), which is the integral of the current density with time. FE-SEM images of Au deposition on the MgOC show that the size of Au particles varied from a few tens of nanometers at 5 C cm^−2^ to 200 nm at 10 C cm^−2^ (Figure 3). We also observed the non-homogeneous growth of Au, indicating the existence of preferential growth sites on the MgOC surface. A cross-sectional view of the electrode modified with gold at 15 C cm^−2^ revealed that most of the deposited gold formed a porous layer on the surface of MgOC (Figure 3f). The porous structure and properties of MgOC may not change throughout the gold electrodeposition process.

In terms of gold adhesion to the MgOc surface, we did not really observe any detachment of the deposited gold nanostructure from the MgOc surface and the adhesion of deposited gold seemed to be of a good quality.

**Figure 4:**
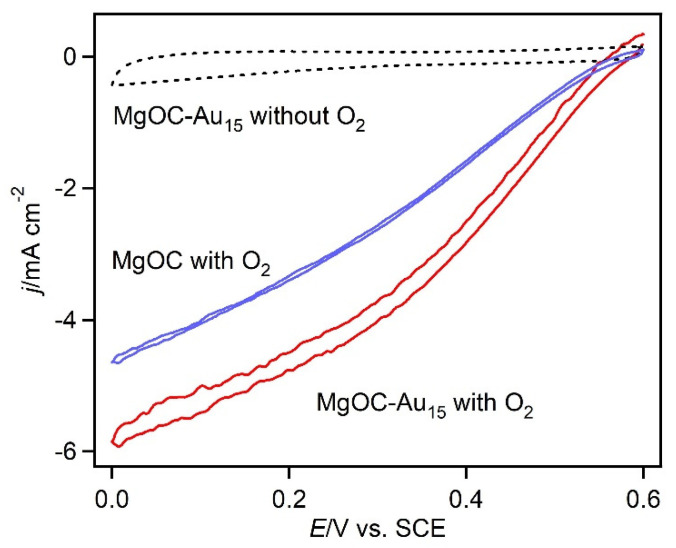
CVs of MgOC without gold nanostructures (blue, O_2_-saturated) and MgOC-Au_15_ (red, O_2_-saturated; dotted, in the absence of O_2_) recorded at 2 mV s^−1^, pH 5.0, and 25 °C, with a citrate buffer (100 mM).

Figure 4 shows the CVs of the BOD-modified MgOC and MgOC-Au_15_ electrodes in an O_2_-saturated 100 mM citrate buffer solution (pH 5) at 2 mV s^−1^. The current curves exhibited a clear sigmoidal catalytic wave under O_2_-saturated conditions. However, the reduction did not reach a plateau and this can be attributed to an ohmic behavior. The onset potential of the MgOC-Au electrode was 570 mV vs. SCE, indicating a direct electrical connection between the electrode and the T1 copper site of the BOD [8,13]. Conversely, an analogous solution through which argon, rather than oxygen, was bubbled showed no reduction current across this potential range. Moreover, the MgOC-Au electrode did not show any ORR current across this potential range in the absence of BOD. These results confirm that the observed ORR currents arise from the direct electron transfer (DET)-type reaction of BOD, while the Au nanostructure exhibits no ORR activity across this potential range. The current density (of −5.9 mA cm^−2^) of the BOD-modified MgOC-Au_15_ at 0 mV was approximately 30% higher than that of the BOD-MgOC electrode (−4.7 mA cm^−2^). The increase in catalytic current does not directly correspond to the increase in surface area (which doubled), implying that the usage efficiency of the gold surface for the BOD redox reaction is lower than that of the carbon surface. This can be improved via surface modification.

### 3.2. Dependence of Amount of Gold on MgOC

**Figure 5:**
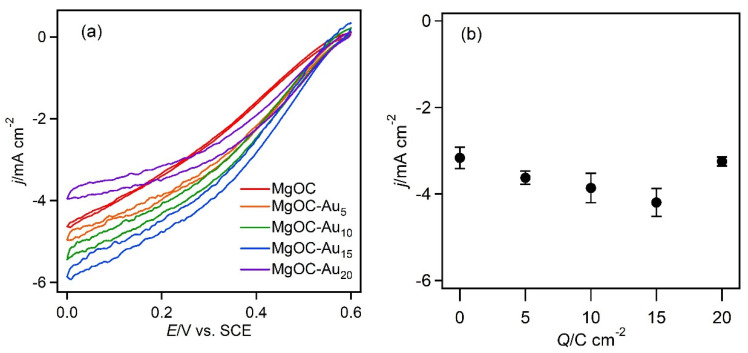
Dependence of the current density on the amount of gold nanostructure deposited on the MgOC electrode: (**a**) CVs and the various electrodes recorded at 2 mV s^−1^, pH 5.0, and 25 °C, with a citrate buffer (100 mM) under O_2_-saturated conditions. (**b**) Current density at 300 s in CA at 0.2 V (pH 5.0, citrate buffer (100 mM), O_2_-saturated, 25 °C).

The current density (Figure 5b) at the MgOC-Au_15_ electrode (−4.2 ± 0.3 mA cm^−2^) was higher than that at the electrode without a gold nanostructure (−3.2 ± 0.2 mA cm^−2^). The catalytic current increased up to the Au deposit corresponding to 15 C cm^−2^, owing to an increase in the surface area and enzyme loading. However, the catalytic current of the MgOC-Au_20_ electrode decreased to −3.2 ± 0.1 mA cm^−2^, implying that Au deposition inhibits mass transfer or changes the nanostructure such that direct electron transfer is disfavored.

### 3.3. Surface Modification of Au on MgOC for Improved Orientation of BOD

To improve the electrical connection between BOD and the gold surface, the orientation of BOD should be such that its T1 redox centers are located as close to the gold surface as possible. The orientation of BOD can be controlled by adjusting the electrostatic interactions during its adsorption onto the electrode surface. The functionalization of the electrode surface with negatively charged aromatic molecules orientates the adsorbed BOD so as to achieve a good electrical connection [14]. While BOD carries a net negative charge, it also has positively charged T1 centers that can interact strongly with the negatively charged gold surface [15].

**Figure 6:**
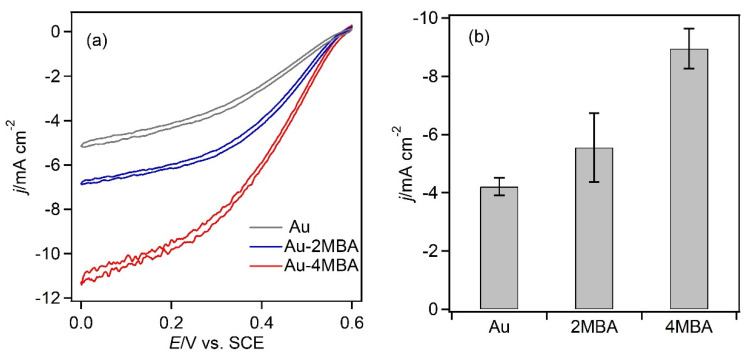
Aromatic thiol-modified MgOC-Au_15_ electrodes. (**a**) CVs of the unmodified (Au, gray), 2MBA-modified (blue), and 4MBA-modified (red) electrodes (recorded under the following conditions: 2 mV s^−1^, pH 5.0, citrate buffer (100 mM), O_2_-saturated, and 25 °C). (**b**) Current density of the unmodified and modified electrodes in CA at 300 s and 0.2 V.

The MgOC-Au_15_ electrode was dipped in 2MBA and 4MBA solutions for 12 h at room temperature, and then washed with distilled water and ethanol to remove the unreacted and adsorbed thiols. The 2MBA-modified electrode showed an ORR current of −5.6 ± 1.0 mA cm^−2^ (Figure 6a), which was 1.3 ± 0.3 times higher than that of the MgOC-Au_15_ electrode (−4.2 ± 0.3 mA cm^−2^). A current density of −8.3 ± 3.0 mA cm^−2^ was obtained using the 4MBA-modified electrode (Figure 6b), which was 1.9 ± 0.7 times higher than that of the MgOC-Au_15_ electrode. The negatively charged carboxyl group at the para position of 4MBA forms attractive electrostatic interactions with the T1 copper without steric hindrance from the aromatic rings or neighboring thiol molecules. Conversely, 2MBA, which has a carboxyl group in the ortho position, is more susceptible to the influence of steric effects. This may explain the higher current density of the 4MBA-modified electrode.

**Figure 7:**
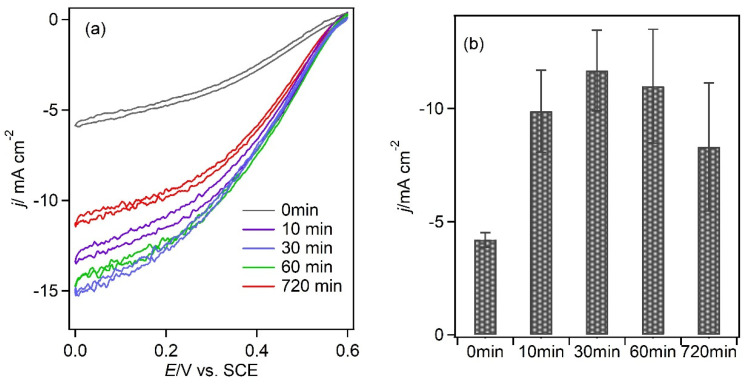
Dependence of the current density on the deposition time of 4MBA on the gold-modified electrode: (**a**) CVs of 4MBA-modifed electrodes obtained after various deposition times (recorded under the following conditions: 2 mV s^−1^, pH 5.0, citrate buffer (100 mM), O_2_-saturated, and 25 °C). (**b**) Current density at 300 s in CA at 0.2 V (under the following conditions: pH 5.0, citrate buffer (100 mM), O_2_-saturated, and 25 °C).

The domain around the T1 copper sites of the BOD is positively charged, whereas the other areas are negatively charged [15]. Accordingly, if the electrode has an excessive negative charge, the current density is reduced owing to the electrostatic repulsion between the electrode and BOD, thereby increasing the distance between them. The surface coverage of 4MBA is an important factor affecting the ORR current. To examine this effect, the coverage of 4MBA on the gold surface was reduced by repeating the modification procedure with deposition times of 10, 30, and 60 min. The CVs (obtained at 2 mV · s^−1^) of the electrodes fabricated with deposition times of 10, 30, 60, and 720 min resulted in ORR currents of −9.9 ± 2 mA cm^−2^, −12 ± 2 mA cm^−2^, −11 ± 3 mA cm^−2^, and −8.3 ± 3 mA cm^−2^, respectively (Figure 7). The highest current density was achieved with a thiol deposition time of 30 min. These results demonstrate that excessive thiol deposition on the electrode surface is detrimental to its performance, which is consistent with the finding that an increased charge changes the structure of BOD [16]. Takahashi et al. reported that the drop-casting of 4MBA-modified gold nanoparticles on Ketjen black yielded an approximately 1.8-fold increase in the ORR current (McIlvaine buffer, pH 5.0, 4 °C, 10 mV s^−1^, 4000 rpm) [17]. In the present study, the current density of the 4MBA-modified electrode fabricated with a deposition time of 30 min was approximately 2.9 ± 0.5 times higher than that of the MgOC-Au_15_ electrode.

## 4. Conclusions

Gold nanostructures were deposited via DHBT on the BOD-based MgOC cathode to increase the effective surface area of the electrode. The observed ORR current density was 1.3 ± 0.1 times greater than that of the electrode without the gold nanostructure. To further improve the ORR current on the gold surface, we controlled the orientation of BOD by modifying the gold-deposited electrodes with aromatic thiol molecules bearing negative charges. The modification time was then optimized. Indeed, the modification of the gold-deposited electrode with 4-mercaptobenzoic acid resulted in a significantly improved ORR current density of 12 mA cm^−2^, four times higher than that of the unmodified MgOC electrode. This indicates that the introduction of aromatic compounds bearing a para-substituent can effectively control the orientation of BOD by modifying the electrostatic interactions with the electrode. These results demonstrate that DHBT is an efficient method for the fabrication of nanostructured electrodes that promote direct electron transfer with oxidoreductase enzymes, such as BOD (Supplementary Materials).

## Data Availability

The data that support the findings of this study are available from the corresponding authors upon reasonable request.

## Supplementary Materials

The following supporting information can be downloaded at: https://www.mdpi.com/article/10.3390/bios13040482/s1. Figure S1. XPs specra of nanostructured gold; Figure S2. Stability curves, under continuous operation, of different biocathodes. Measurement performed at 200 mV vs SCE, pH 5.0, and 25 °C, with a citrate buffer (100 mM).
