# Supplementary material for: Hierarchical Structure of Gold and Carbon Electrode for Bilirubin Oxidase-Biocathode
Source: Biosensors (Basel). 2023 Apr 17;13(4):482. doi: 10.3390/bios13040482 (PMC10136233; doi:10.3390/bios13040482)
Supplement: Supplementary file 1 [file biosensors-13-00482-s001.zip › biosensors-2269243-supplementary.pdf]

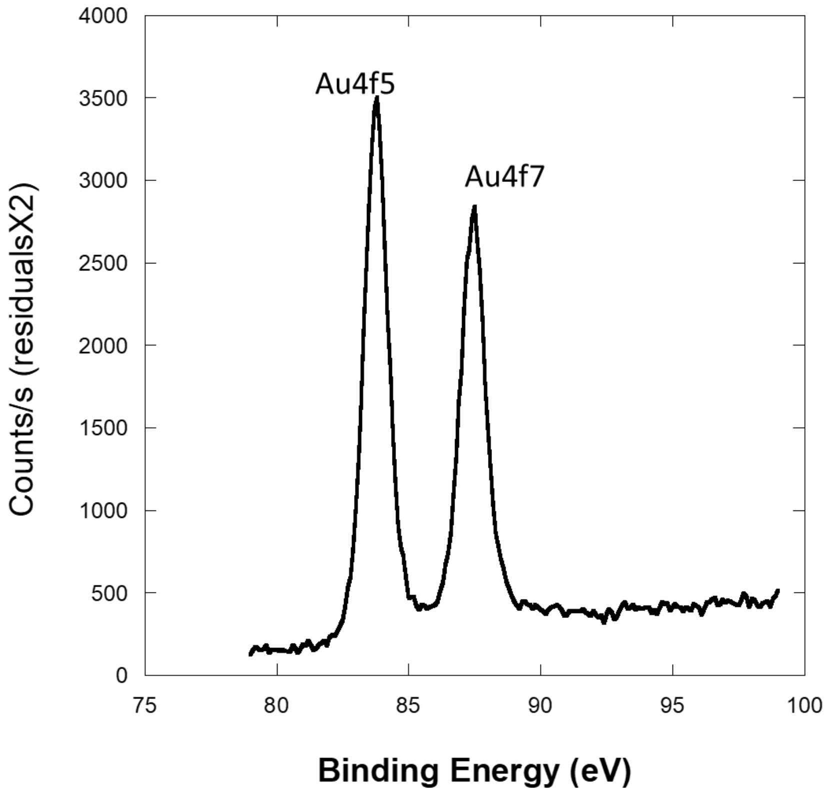

Figure S1. XPs specra of nanostructured gold

Figure S2 shows the chronoamerometry response, during 25 hours, at 200 mV, of BOD modified of MgOC, Au-MgOC, and 4MBA modified Au-MgOC electrodes. We cannot see any difference in the slope, although initial currents are different. from this result, the stability is basically determined by the enzyme itself, and the enzyme is not affected by the surface (not denaturated and stabilized).

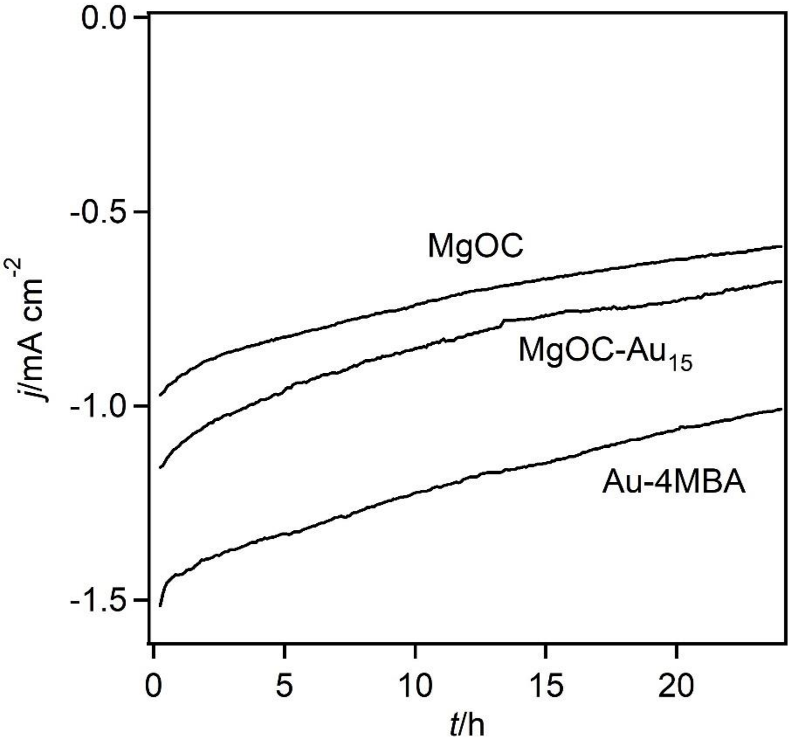

Figure S2. Stability curves, under continuous operation, of different biocathodes. Measurement performed at 200 mV vs SCE, pH 5.0, and 25 °C, with a citrate buffer (100 mM).
